# HIV False-Positive Test in the Setting of CD4 Lymphocytopenia

**DOI:** 10.7759/cureus.51515

**Published:** 2024-01-02

**Authors:** Hussien Mohamed, Hanna D Hedriana, Emily A Holbrook, Heather Henderson, Jason W Wilson

**Affiliations:** 1 Emergency Medicine, Christiana Care, Newark, USA; 2 Emergency Medicine, Lakeland Regional Hospital, Lakeland, USA; 3 Anthropology, University of South Florida, Tampa, USA; 4 Emergency Medicine, University of South Florida Health Morsani College of Medicine, Tampa, USA

**Keywords:** false-positive hiv, false positive, cdc hiv algorithm, cd4 lymphocytopenia, false hiv test, hiv testing

## Abstract

In 2016, we implemented a non-targeted Emergency Department (ED)-based HIV screening program at our academic medical center following revised CDC guidelines utilizing the Abbott Alinity 4th generation HIV-1/2 antigen (Ag)/antibody (Ab) immunoassay (Abbott Laboratories, Abbott Park, IL). Following the CDC algorithm, after reactive fourth-generation testing, HIV-1/2 Ab testing is conducted. Patients undergoing acute seroconversion (acutes) may express p24 Ag but have a negative confirmatory Ab test. Acutes have the same laboratory signature during the ED encounter as those that are false positive (False +), and the two patient groups are denoted as “equivocals” until viral load testing specifies a definitive HIV status. Among False + patients (Ab/Ag positive, Ab negative, viral load undetectable), there have been limited studies on those also demonstrating a reduction in CD4+ count, an uncommon phenomenon known as “idiopathic CD4 lymphocytopenia.” We review a patient with a reactive fourth-generation HIV Ab/p24 Ag test on two separate occasions. Despite lymphopenia with a reduced CD4 count, his symptoms resolved, and an RNA PCR test did not detect any presence of HIV (False +). This patient was unique as False + patient with p24 Ag reactive, as well as a coincidental low CD4 count in the absence of HIV infection. A low CD4 count is often a sign of significant HIV infection.

## Introduction

During an Emergency Department (ED) encounter that encompasses non-targeted HIV screening, patients that have a reactive HIV-1/2 Ab/p24 Ag screening assay then undergo confirmatory HIV-1/2 Ab testing following the revised CDC algorithm [1-2]. We have observed that 10% of patients in the acute encounter will have a reactive Ab/Ag screening test, but no HIV Ab on confirmatory testing. This equivocal (“equivocals”) test signature can be the result of an acute HIV infection (“acutes”) or a false-positive (“false positives”) test secondary to presence of p24 Ag. Acutes are patients tested prior to mounting an Ab response while undergoing acute seroconversion. False positives are patients that have p24 Ag for reasons other than HIV. Published case reports have shown several reasons a patient may test false positive, including lupus, CAR T-cell therapy, rheumatoid arthritis, and other autoimmune diseases [3-5]. Resolution of equivocals requires HIV RNA PCR testing to look for definitive presence of virus.

HIV RNA PCR nucleic acid testing has a typical clinical turnaround time of 72 hours secondary to batching to limit reagent waste. The majority of patients that are HIV Ab/Ag reactive will be true positives. For these two reasons, adjunct labs, including CD4+ count, are often obtained at the point of an HIV Ab/Ag reactive screening test. The absolute CD4+ helps stratify disease progression. HIV negative patients typically do not have reduced CD4+ levels. There is limited research on false-positive patients (HIV Ag/Ab reactive, HIV Ab negative, no detectable HIV on RNA PCR) who also have a reduction in their CD4+ lymphocytic count.

One reason for this may be the small number of cases reported with p24 antigen positivity and CD4+ lymphocytopenia, without the presence of an HIV infection. Prior authors have labeled this phenomenon as “idiopathic CD4 lymphocytopenia” [6-8]. Others have documented CD4 reduction in cases of malaria, viral, bacterial, and parasitic infections, psychological stress, burns, malnutrition, corticosteroid use, over-exercising, pregnancy, and normal variation [9]. However, in those scenarios, CD4+ count is not part of the initial diagnostic and risk stratification approach to new infection as in HIV. We report a case of a patient who was reactive for the p24 antigen on two separate fourth-generation HIV-1/2 Ag/Ab screening tests [10] with subsequent negative antibody and no detectable HIV on RNA PCR testing, coupled with a profound reduction of CD4+ lymphocytes.

## Case presentation

A 45-year-old male with eosinophilic esophagitis (EOE) and previous negative HIV test six years ago presented to the ED with the chief complaint of left facial swelling, weakness, and numbness (Table 1). He started to notice the symptoms about one hour after he woke up but was unsure of when the symptoms began. The numbness/swelling/weakness started on his left upper lip and spread to his nose and cheek (V2 distribution). After arriving to the hospital, the weakness slightly improved. He denied any associated facial droop, dysarthria, dysphagia, tinnitus, auditory symptoms, blurred vision, or headache. He reported no history of stroke or TIA. He denied any recent trauma, bug bites, fever or chills. Of note, the patient also self-reported that he was recovering from a gastrointestinal infection three days prior. His medications included oral budesonide for EOE. He has no pertinent family history, and he denies tobacco, alcohol, and illicit drug use. While in the ED, he developed pruritus and hives along his left lateral thigh and arm that resolved with methylprednisolone and famotidine. He received no medications in the ED prior to urticaria eruption (Table 1).

**Table 1:** Demographics, medical history, diagnostic results, and laboratory values. ED = Emergency Department NIHSSS = NIH Stroke Scale Score PCR = Polymerase Chain Reaction

Demographic Variable, Medical Finding, or Laboratory Measurement	Result/Value
Age	45
Biological Sex	Male
Past Medical History	Eosinophilic Esophagitis (EOE)
Pertinent History	Negative HIV test 6 years prior to encounter

ED Chief Complaint	Left facial swelling, weakness, numbness X 1 hour after waking up; V2 distribution
ED/Hospital Course	Developed urticaria in the ED; all other symptoms resolved on hospital day 2
Medications in ED	Methylprednisolone and famotidine
NIHSSS	1
CT Brain without Contrast	Right frontal encephalomalacia, No acute findings
MRI Brain without Contrast	Encephalomalacia of the posterior right frontal lobe
White Blood Cell Count	3.23 x 103/uL [5,000-10,000 103/uL]
Absolute CD4 Count, % Lymphocyte Count	230 cells/mm^3^ [500-1,500 cells/mm^3^], 28% [30%-60%]
Serum HIV Ab/p24 Ag Screening Test #1 via Abbott ARCHITECT	Reactive
HIV RNA PCR #1 via Nucleic Acid Testing	No virus detected
Serum HIV Ab/p24 Ag Screening Test #2 via Abbott ARCHITECT	Reactive
HIV-1, HIV-2 Ab Test #2 via Bio-Rad Geenius	Not detected
HIV RNA PCR Nucleic Acid Testing via the Hologic Aptima	No virus detected

**Figure 1:**
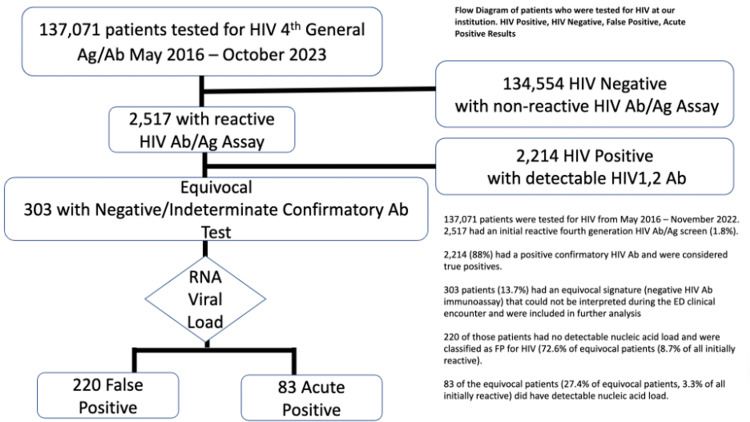
Flow diagram of patients who have undergone ED-based HIV screening at one academic medical center based on revised CDC guidelines.

In the ED, his vitals remained stable, and he had a NIH stroke scale score (NIHSSS) of 1. His CT head without contrast showed evidence of right frontal encephalomalacia (Table 1). The patient was screened for HIV due to implementation of an ED-based non-targeted HIV screening program following CDC guidelines (Figure 1). Inclusion criteria include people aged 13-64 years of age with no evidence of having the fourth generation HIV screening test within the past year in our electronic medical records (EMR). His fourth generation HIV Ab/p24 Ag screening test was reactive on two separate occasions on the Abbott ARCHITECT [10]. His follow-up ‘‘confirmatory’’ HIV-1 and HIV-2 antibody test was negative on the Bio-Rad Geenius [11]. An order was then placed for an HIV viral load via RNA PCR on the Hologic Aptima [12]. His white blood count (WBC) was 3.23 x 10^3^/uL (5,000-10,000 x 10^3^/uL), but his complete blood count and complete metabolic panel were otherwise unremarkable. Due to the speculation of a possible HIV infection, a CD4+ count was ordered, which resulted in an absolute CD4+ count of 230 cells/mm^3 ^(500-1,500 cells/mm^3^), making up 28% of the lymphocytic count (30%-60%) (Table 1). Due to these findings, the patient was admitted for further evaluation, including magnetic resonance imaging (MRI) and neurology consult.

MRI showed encephalomalacia of the posterior right frontal lobe. The patient had symptom resolution the following day, and the RNA PCR test did not detect any presence of HIV (<30 IU/mL). His HIV testing was subsequently labeled as a false positive despite having CD4+ lymphocytopenia. The patient was subsequently discharged due to resolution of symptoms.

## Discussion

We present a case of a patient who had a false-positive test result for HIV on two separate occasions (HIV Ab/Ag reactive, HIV-1/2 Ab negative, no detectable HIV via RNA PCR). There are multiple reasons to explain a reactive HIV with the fourth-generation HIV Ab/p24 Ag test, including having a p24 antigen, HIV-1 or HIV-2 antibodies in the system, or possible test error.

In this case, the patient underwent fourth-generation serum testing on two different samples during the hospital course. Each time, the patient had a reactive p24 antigen. This fundamentally rules out the possibility of a lab or test error. Since the patient tested negative for both HIV-1 and HIV-2 antibodies each time, the only remaining reason for having a false-positive result twice could be due to the presence of p24 antigen in his blood system (Figure 1). It is important to note that his CD4+ count was concurrently low, along with having a p24 antigen, which has been reported at least once by the CDC in 1989 [13]. The previous patient reported by the CDC had a p24 antigen verified via Western blot and a CD4+ count of 103. These cases pose important questions for future investigation: does the p24 antigen have any association with the CD4+ counts or are these isolated events? Do these patients simply have idiopathic CD4 lymphocytopenia and another reason for having a p24 antigen not yet discovered? To answer these questions, we will need more research and awareness of these occurrences. We do speculate that, if we tested everyone with idiopathic CD4 lymphocytopenia for the p24 antigen, we may see more positive test results, as evidenced by the patient in this case.

## Conclusions

This case shines light on clinically false-positive HIV tests (HIV Ab/Ag reactive, HIV Ab/Ag negative, no detectable HIV on RNA PCR testing), discovering low CD4 counts occurring in the absence of an HIV infection, and investigates the link between the p24 antigen and low CD4 counts. The identical laboratory signature of equivocals, including false positive and acute seroconversion during the ED encounter, represents two opportunities for improved clinical scenarios. The lack of available rapid HIV RNA PCR leads to stress and unnecessary harm among patients who are p24 reactive and HIV Ab negative, but HIV RNA PCR negative. On the other hand, this lack of rapid nucleic acid test availability leads to missed opportunities to link patients to care, initiate treatment, and decrease HIV transmission in those who are p24 reactive, HIV Ab negative, and HIV RNA PCR positive during an important window among those with new HIV infections. In patients with initial fourth-generation serum HIV reactivity, but negative confirmatory HIV Ab testing, providers should consider the entire clinical picture before discussing the results with the patient. For example, is the patient a person who injects drugs, someone recently incarcerated, a sex worker, or a man who has sex with men? Does the chief complaint mirror a viral prodrome (e.g., fever, sore throat, cough, malaise, etc)? This phenotypic context can help guide the conversation that, ultimately, an RNA result will be needed to formally diagnose or rule out HIV.

CD4+ counts may not contribute to distinguishing HIV status after reactive screening while awaiting RNA PCR testing, given the possibility of low CD4+ counts among those with p24 antigen. In this case, the patient underwent unnecessary stress when a possible diagnosis of HIV disclosed. However, fortunately, it uncovered the CD4+ lymphocytopenia as CD4 counts are not initially ordered unless you speculate a potential HIV infection. Research has shown that low CD4 counts could be due to multiple reasons, including idiopathic CD4 lymphocytopenia, infections, pregnancy, etc. However, there has been little evidence of its association with the p24 antigen. More research and awareness of this possible correlation need to be considered to help guide ED physicians in their decisions to order a CD4 count when patients are identified as false positive for HIV due to the systemic presence of p24 antigen.
